# FIGO position statement on the use of the WHO labor care guide versus the partograph

**DOI:** 10.1002/ijgo.70151

**Published:** 2025-04-26

**Authors:** Akaninyene E. Ubom, Albaro J. Nieto‐Calvache, Zechariah J. Malel, Inês Nunes, FIGO Committee on Childbirth and PPH

**Affiliations:** ^1^ Department of Obstetrics, Gynecology and Perinatology Obafemi Awolowo University Teaching Hospitals Complex Ile‐Ife Nigeria; ^2^ Departamento de Ginecología y Obstetricia Fundación Valle del Lili Cali Colombia; ^3^ Faculty of Health Sciences Universidad ICESI Cali Colombia; ^4^ Department of Obstetrics and Gynecology, School of Medicine University of Juba Juba South Sudan; ^5^ Association of Gynecologists and Obstetricians of South Sudan (AGOSS) Juba South Sudan; ^6^ Department of Obstetrics and Gynecology Gaia/Espinho Local Health Unit Porto Portugal; ^7^ CINTESIS—Center for Health Technology and Services Research University of Porto Porto Portugal; ^8^ Department of Medical Sciences University of Aveiro Aveiro Portugal

**Keywords:** labor, labor care guide, partograph, World Health Organisation

## Abstract

The partograph, a graphical tool for recording labor events and assessing progress, was initially introduced by Dr. Emmanuel Friedman in 1954. Subsequent development by Philpott and Castle led to the widely adopted partograph in 1972. The World Health Organisation (WHO) endorsed its use in all labor wards in 1994, based on a multicenter trial demonstrating a significant reduction in prolonged labor, labor augmentation, emergency caesarean sections, and intrapartum stillbirths. In 2018, the WHO updated its global recommendations on intrapartum care, prompting a revision of the partograph. This resulted in the 2020 launch of the WHO Labor Care Guide (LCG), a supplementary monitoring tool. The LCG comprises seven sections, focusing on information at admission, supportive care, maternal and fetal care, labor progress, medication, and shared decision‐making. Unlike the classic partograph, the LCG considers the active phase of labor to begin at a cervical dilatation of 5 cm. This evolution reflects ongoing efforts to improve maternal and fetal outcomes through evidence‐based labor management. In this position statement FIGO recommends that the WHO LCG should be universally adopted and used to monitor labor in all settings to reduce maternal and perinatal mortality, and to support the implementation of the WHO recommendations: intrapartum care for a positive childbirth experience.

## 1 BRIEF HISTORY OF THE PARTOGRAPH

The partograph is a universal tool used to graphically record labor events, facilitating the easy assessment of labor progress and its management. The history of the partograph dates back to 1954, when Emmanuel Friedman introduced the cervicograph, a plot of cervical dilatation against time, which he had recorded for 100 primigravid women.^1^ Based on Friedman's cervicograph, Philpott and Castle developed the first partograph in 1972.^2^ In 1994, as part of the safe motherhood initiative that was launched in Nairobi in 1987, the WHO revised, approved, and recommended the partograph to be used in all labor wards to reduce maternal and fetal mortality.^3^ The recommendation was based on the results of a multicenter trial involving 35 484 women, which showed that the use of the partograph in the management of labor reduced the incidence of prolonged labor (6.4%–3.4%), labor augmentation (20.7%–9.1%), emergency cesarean sections (9.9%–8.3%), and intrapartum stillbirths (0.5%–0.3%).^3^

## 2 THE WHO LABOR CARE GUIDE

In 2018, the WHO published its updated global recommendations on intrapartum care, which included new definitions of the duration of the first and the second stages of labor, and provided guidance on the timing and use of labor interventions to improve the health and wellbeing of laboring women and their babies.^4^ Following this update, the WHO initiated the process of revising the partograph in line with the current evidence. As a result, a new labor monitoring tool called the WHO Labor Care Guide (LCG) (Figure S1) was launched in 2020.^5^ The LCG has seven sections: (1) Identifying information and labor characteristics at admission, (2) supportive care, (3) care of the baby, (4) care of the woman, (5) labor progress, (6) medication and (7) shared decision making. In contrast to the classic partograph, in which the active phase of labor starts at a cervical dilatation of 4 cm, the starting point of the active first stage of labor in the LCG is a cervical dilatation of 5 cm. In addition, the minimum rate of cervical dilatation that is considered normal in the LCG is 0.5 cm/h and not the 1 cm/h upon which the design of the previous partograph was based. WHO premised this change on the fact that a minimum cervical dilatation rate of 1 cm/h throughout the active first stage of labor has been found to be unrealistically fast for some women.^6^

## 3 FIGO'S OBSERVATIONS AND POSITION ON THE USE OF THE PARTOGRAPH VERSUS THE LCG

It is the intention of the WHO that the LCG replaces the partograph. FIGO supports this position based on current best evidence, which informed the revision of the previous partograph and the design of the LCG in the first instance. However, FIGO anticipates that a replacement of the widely accepted partograph, which has been in use for more than half a century, with the new LCG, may provoke anxiety, apathy, uncertainty, and even opposition among labor care providers globally. FIGO acknowledges that save for its appearance, the core concept and characteristic of the partograph, which is the graphical representation of labor progress in terms of the woman's cervical dilatation and descent of the fetal presenting part against time, has not changed and retains its key position in the LCG. Regular recordings of important clinical maternal and fetal parameters, as well as medications, including oxytocin and intravenous fluids, have also been retained in the LCG.

FIGO notes various advantages of the LCG over the partograph. The LCG has an “alert” column, which presents thresholds for abnormal labor observations that require further assessment and action by the labor care provider. Any abnormal observation is circled, to help highlight these abnormal observations for prompt and timely interventions. In addition, the LCG addresses an important deficit of the classical partograph by including the second stage of labor, emphasizing continued monitoring of the mother and fetus during this stage. The LCG also emphasizes respectful maternity care by explicitly requiring recording of supportive care in labor, including companionship, and fluid and analgesic management, as well as maternal position and mobility. The shared decision‐making section of the LCG aims to facilitate continuous communication of care and interventions with the woman and her companion, and the consistent recording of all assessments and plans/decisions agreed. The use of the LCG has been demonstrated to be feasible and acceptable in different clinical settings, with the potential to promote woman‐centered care.^7^ In a large trial involving 26 331 participants, the LCG reduced the cesarean section rate by 5.5% and labor augmentation by 18%.^8^

Heavy workloads in many labor wards may, however, make it challenging to consistently and accurately record all the observations in the LCG.^7^ This challenge is not unique to the WHO's LCG but also applies to the partograph. The physical layout of many labor wards may not support the implementation of certain LCG components, such as companionship. In addition, poor staffing and limited equipment and medication supplies pose further challenges in many settings. Being a relatively new tool, the LCG is understandably not widely available yet. Therefore, many labor care providers may not be familiar with or trained in its use. FIGO also recognizes that significant additional efforts are required for the LCG to be widely implemented. Support from scientific societies and local health oversight organizations is essential during the implementation process in local hospitals. Furthermore, medium‐ and long‐term follow‐ups are necessary to ensure its proper and sustained use.

## 4 FIGO RECOMMENDATIONS

1. The WHO's LCG should be universally adopted and used to monitor labor in all settings to reduce maternal and perinatal mortality, and to support the implementation of *WHO Recommendations: Intrapartum Care for a Positive Childbirth Experience*.
2. Health systems should give every labor care provider adequate training on the use of the WHO LCG to ensure its effective use.
3. National and hospital policies should guarantee that all labor wards facilities, equipment, and medical supplies are updated to provide an enabling environment for the effective implementation of the WHO LCG.
4. Labor wards should be adequately staffed to ensure that every observation in the WHO LCG is consistently and accurately recorded for all women in labor, to minimize errors and prevent labor complications.
5. All laboring women should be allowed companions, as stipulated in the LCG. This will promote respectful maternity care, enable women's autonomy, and improve maternal and perinatal outcomes.
6. Health systems, through quality improvement teams in hospitals, should champion the introduction of the LCG into the hospitals, adequately supervise and conduct regular audits to guarantee that the WHO LCG is consistently, accurately, and efficiently used to monitor all labors, and to ensure its sustainability.
7. In settings where the WHO LCG is unavailable or staff have not yet been trained in its use, the partograph should continue to be used for labor monitoring until conditions allow for its replacement with the WHO LCG.

## 5 FIGO COMMITMENTS

FIGO is committed to effectively decrease maternal and perinatal morbidity and mortality from labor and delivery.

FIGO will support the efforts of our member societies, regional federations, healthcare colleagues, and policy makers to ensure the global adoption and use of the WHO LCG.

FIGO will support clinical and administrative stakeholders, national and hospital policy makers, through her member societies, to ensure adequate staffing and update/upgrade the labor wards' facilities, equipment, and medical supplies, to provide an enabling environment for the effective implementation of the WHO LCG globally. FIGO will collaborate with its member societies to routinely measure progress in these regards.

## AUTHOR CONTRIBUTIONS

Article planning and literature review: A.E.U., I.N. Manuscript editing: A.E.U., A.J.N.‐C., Z.J.M., I.N. All authors read and approved the final manuscript.

## FUNDING INFORMATION

The authors received no funding for this position statement.

## CONFLICT OF INTEREST STATEMENT

The authors have no conflicts of interest.

## Supporting information


Figure S1

## Data Availability

Data sharing is not applicable to this article as no new data were created or analyzed in this study.
