# Supplementary figures and images for: FIGO position statement on the use of the WHO labor care guide versus the partograph
Source: Int J Gynaecol Obstet. 2025 Apr 26;170(1):25–7. doi: 10.1002/ijgo.70151 (PMC12177285; doi:10.1002/ijgo.70151)

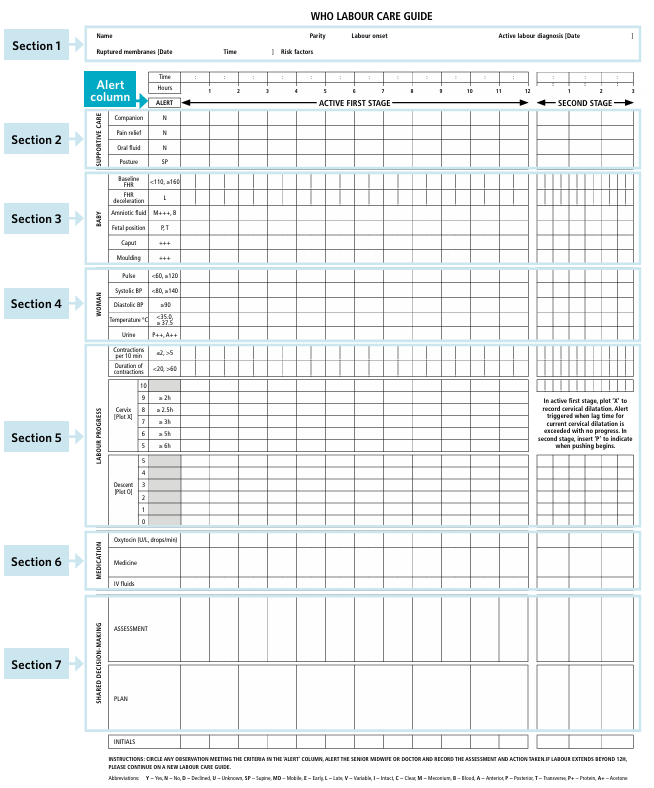

Supplement: Supplementary file 1 — Figure S1 [file IJGO-170-25-s001.png]
